# Supplementary material for: Effect of Hydroxymethylfurfural and Low-Molecular-Weight Chitosan on Formation of Acrylamide and Hydroxymethylfurfural during Maillard Reaction in Glucose and Asparagine Model Systems
Source: Polymers (Basel). 2021 Jun 8;13(12):1901. doi: 10.3390/polym13121901 (PMC8229482; doi:10.3390/polym13121901)
Supplement: Supplementary file 1 [file polymers-13-01901-s001.zip › polymers-1249863-SI.pdf]

Supplementary material

(A)

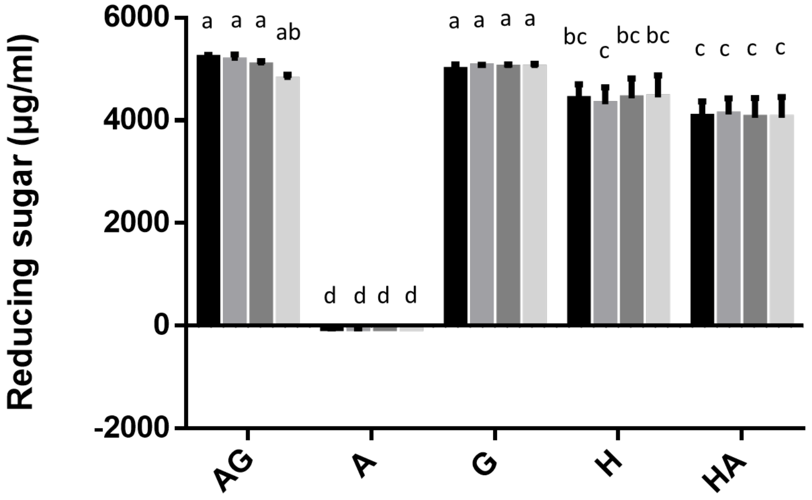

(B)

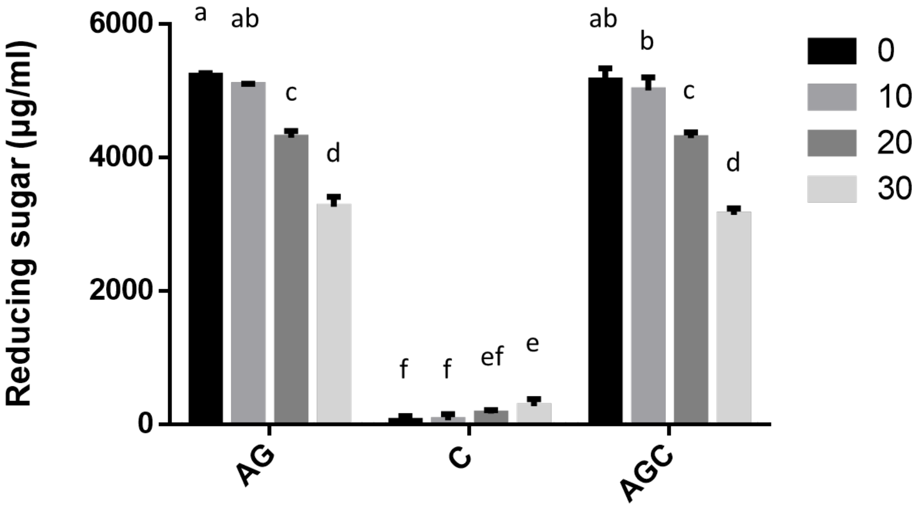

Figure S1. Amounts of reducing sugar between different groups in (A) deionized water (B) acetic acid at various time points during heating processings.. AG: Asparagine + Glucose; A: Asparagine; G: Glucose; H: Hydroxymethylfurfural; HA: Hydroxymethylfurfural + Asparagine; C: Chitosan; AGC: Asparagine + Glucose + Chitosan. Values are expressed as mean ± standard deviation (SD) (n = 3). [a-f] Indicate significant differences between different groups ($p < 0.05$).

(A)

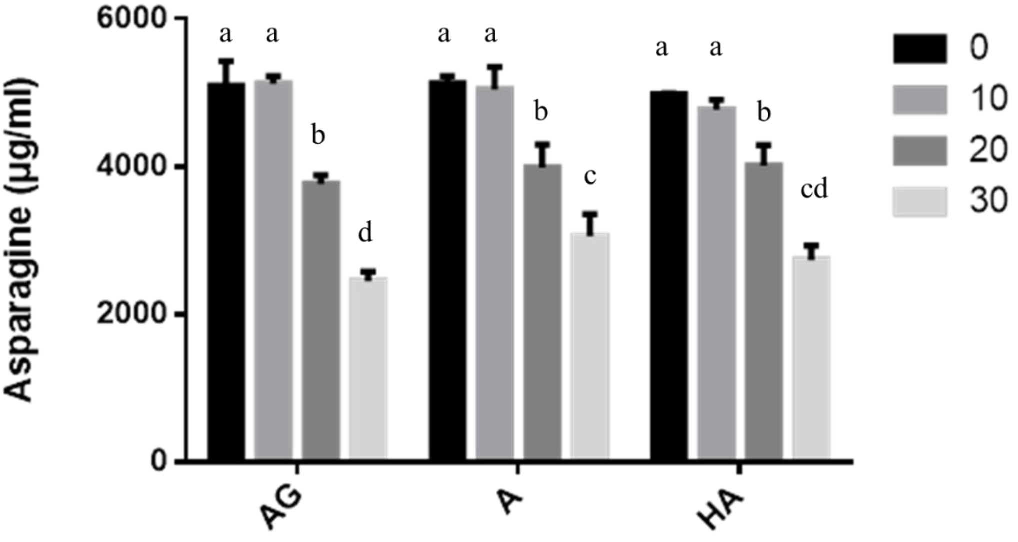

(B)

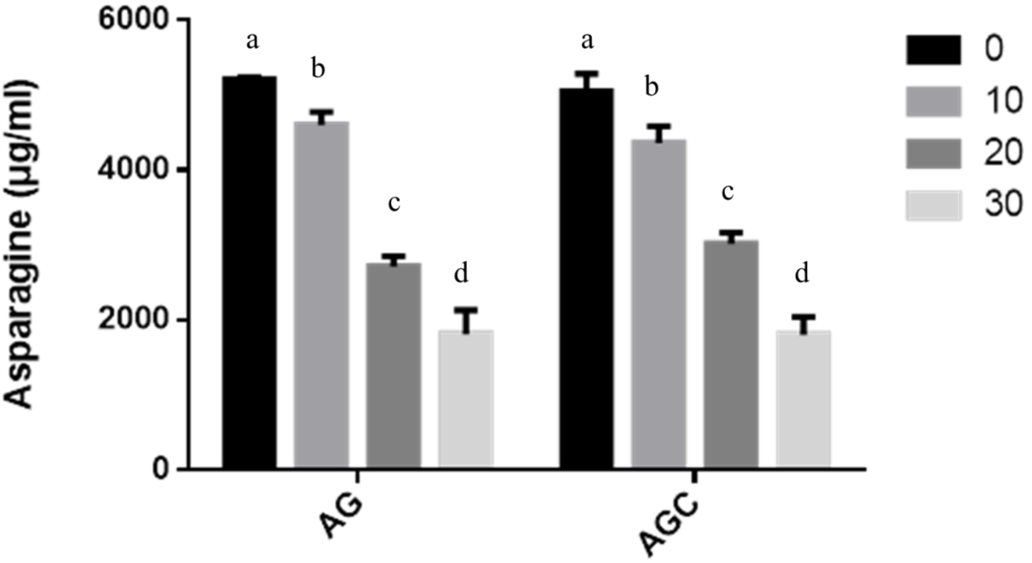

Figure S2. Amounts of asparagine between different groups in (A) deionized water (B) acetic acid at various time points during heating processings. AG: Asparagine + Glucose; A: Asparagine; HA: Hydroxymethylfurfural + Asparagine; AGC: Asparagine + Glucose + Chitosan. Values are expressed as mean ± standard deviation (SD) (n = 3). [a-e] Indicate significant differences between different groups ($p < 0.05$).

(A)

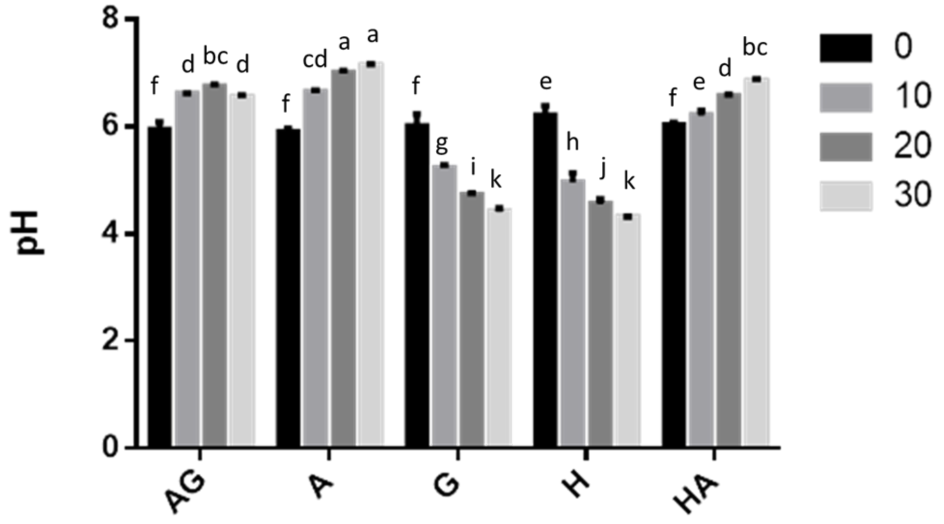

(B)

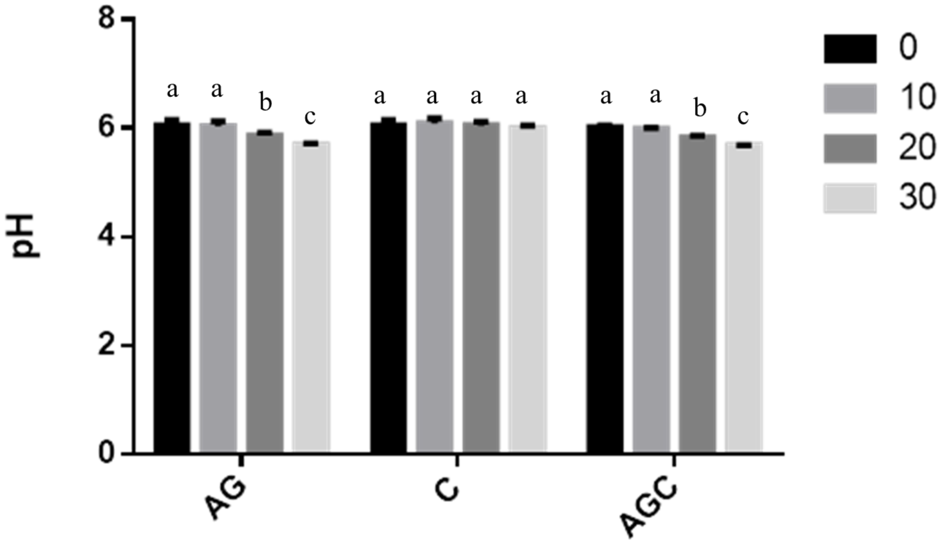

Figure S3. The pH values between different groups in (A) deionized water (B) acetic acid at various time points during heating processings. AG: Asparagine + Glucose; A: Asparagine; G: Glucose; H: Hydroxymethylfurfural; HA: Hydroxymethylfurfural + Asparagine; C: Chitosan; AGC: Asparagine + Glucose + Chitosan. Values are expressed as mean ± standard deviation (SD) (n = 3). [a-k] Indicate significant differences between different groups ($p < 0.05$).

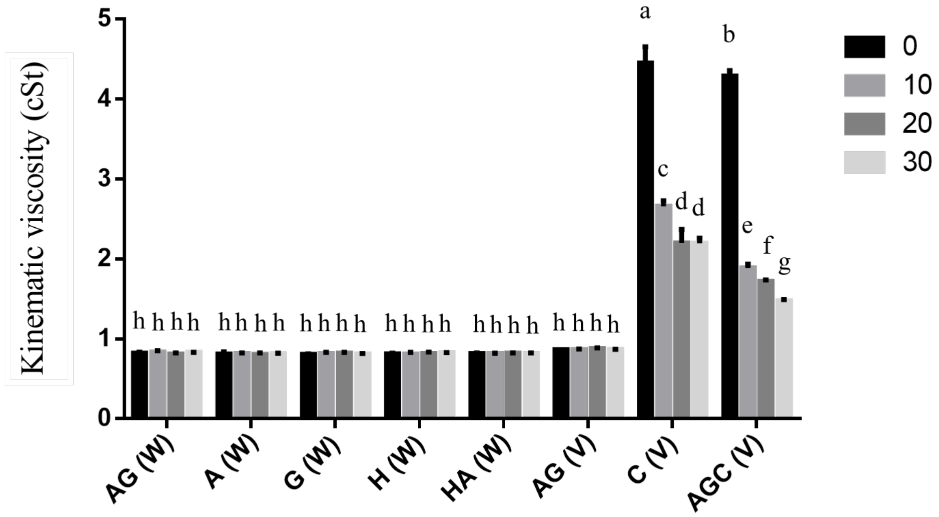

Figure S4. Kinematic viscosity (cSt) between different groups. ID water: deionized water; V: acetic acid; AG: Asparagine + Glucose; A: Asparagine; G: Glucose; H: Hydroxymethylfurfural; HA: Hydroxymethylfurfural + Asparagine; C: Chitosan; AGC: Asparagine + Glucose + Chitosan. Values are expressed as mean ± standard deviation (SD) (n = 3). [a-h] Indicate significant differences between different groups ($p < 0.05$).
